# Irradiated Tumor Fibroblasts Avoid Immune Recognition and Retain Immunosuppressive Functions Over Natural Killer Cells

**DOI:** 10.3389/fimmu.2020.602530

**Published:** 2021-01-22

**Authors:** Nannan Yang, Kristin Lode, Rodrigo Berzaghi, Ashraful Islam, Inigo Martinez-Zubiaurre, Turid Hellevik

**Affiliations:** ^1^Department of Community Medicine, Faculty of Health Sciences, UiT — The Arctic University of Norway, Tromsø, Norway; ^2^Department of Clinical Medicine, Faculty of Health Sciences, UiT—The Arctic University of Norway, Tromsø, Norway; ^3^Department of Radiation Oncology, University Hospital of Northern Norway, Tromsø, Norway

**Keywords:** tumor microenvironment, TME, ionizing radiation, radiotherapy, immunotherapy, immune evasion, immunosuppression, cancer-associated fibroblasts

## Abstract

Recent studies have demonstrated that radiotherapy is able to induce anti-tumor immune responses in addition to mediating direct cytotoxic effects. Cancer-associated fibroblasts (CAFs) are central constituents of the tumor stroma and participate actively in tumor immunoregulation. However, the capacity of CAFs to influence immune responses in the context of radiotherapy is still poorly understood. This study was undertaken to determine whether ionizing radiation alters the CAF-mediated immunoregulatory effects on natural killer (NK) cells. CAFs were isolated from freshly resected non-small cell lung cancer tissues, while NK cells were prepared from peripheral blood of healthy donors. Functional assays to study NK cell immune activation included proliferation rates, expression of cell surface markers, secretion of immunomodulators, cytotoxic assays, as well as production of intracellular activation markers such as perforin and granzyme B. Our data show that CAFs inhibit NK cell activation by reducing their proliferation rates, the cytotoxic capacity, the extent of degranulation, and the surface expression of stimulatory receptors, while concomitantly enhancing surface expression of inhibitory receptors. Radiation delivered as single high-dose or in fractioned regimens did not reverse the immunosuppressive features exerted by CAFs over NK cells *in vitro*, despite triggering enhanced surface expression of several checkpoint ligands on irradiated CAFs. In summary, CAFs mediate noticeable immune inhibitory effects on cytokine-activated NK cells during co-culture in a donor-independent manner. However, ionizing radiation does not interfere with the CAF-mediated immunosuppressive effects.

## Introduction

Technological advances introduced in image-guidance, organ motion management, treatment technique, and radiation delivery have given radiation oncologists the ability to deliver highly conformal, high-dose radiation over fewer fractions, a modality known as stereotactic body radiotherapy (SBRT) (1). In parallel, recent research efforts have focused on the complex interplay between radiation therapy (RT) and the immune system. This has led to the recognition that therapeutic effects of RT, especially when delivered in high-dose hypo-fractionated regimens, may depend on antitumor immune responses in addition to the well-characterized DNA damage-based mechanisms. In line with these ideas, the ability of radiotherapy to induce synergistic responses in partnership with immunotherapy has recently gained widespread interest (2, 3) and currently constitute an attractive option for treating locally advanced non-small cell lung cancers (NSCLCs) (4, 5).

The reactive stroma of solid tumors consists of malignant cells and an ample collection of non-transformed cells including immune cells, mesenchymal cells, pericytes, blood- and lymphatic endothelial cells, as well as signaling molecules and structural proteins. Cancer-associated fibroblasts (CAFs) represent a dominant cell type of the tumor stroma and their presence in large numbers is frequently correlated with extensive desmoplasia, treatment resistance, and poor outcomes (6). The role of CAFs as promoters of tumor growth, invasion, and metastasis is facilitated by their capacity to orchestrate tumor-related inflammation and cellular crosstalk. In contrast to quiescent normal tissue fibroblasts, the heterogeneous population of CAFs possesses the common trait of being synthetically active, displaying enhanced secretion of cytokines, growth factors, proteases, and extracellular matrix (ECM) components, in addition to exhibiting higher proliferation and migration rates (6, 7). As major constituents of the tumor stroma, CAFs participate actively in the regulation of both innate and adaptive anti-tumor immune responses (8). In fact, through the secretion of a plethora of immunoregulatory signal molecules, stromal fibroblasts are efficient regulators of the local immunity in tumors, with the capacity to directly affect trafficking, state of differentiation, and activation of a broad population of immune cells (8).

In the context of radiation, CAFs are known to be highly radioresistant and may survive even ablative doses of ionizing radiation (1x18 Gy) (9, 10). In culture conditions, exposure to medium or high doses of ionizing radiation (IR) does not trigger immunogenic cell death in CAFs (11), but elicit permanent DNA damage responses and a concomitant senescence state accompanied by functional changes, e.g. reduction of proliferation, migration, and invasion rates (9). Radiation-induced changes have also been observed in the cell secretome and paracrine signaling processes mediated by CAFs (12). Of note, earlier *in vitro* studies have suggested that the immunoregulatory effects of CAFs on T cells remain unchanged after exposure to radiation (11). Likewise, CAFs seem to maintain their immunosuppressive effects on M1 macrophages after irradiation (13). Our group has earlier demonstrated that irradiated CAFs may lose their pro-tumorigenic potential *in vivo* in mice after mixed cell transplantations (14). Other groups have reported that irradiated CAFs enhance the invasiveness of pancreatic cancer cells (15) and esophageal squamous cell carcinoma cells (16). Moreover, several studies have shown that CAFs contribute to radiotherapy resistance (17–20), promote irradiated-cancer cell recovery and tumor recurrence post-radiation through the autophagy pathway (20). These findings support the notion that radiation regulates the pro-tumorigenic ability of CAFs. Although it is well established that CAFs play important roles in anti-tumor immune responses, knowledge on the crosstalk between CAFs and immune cells during and/or after radiotherapy remain scarce.

Natural killer cells (NK cells) are innate effector cells with a natural ability to kill virus-infected cells and tumor cells (21), and also produce cytokines and communicate with other immune cells (21, 22). NK cells’ lytic functions are regulated by stimulatory and inhibiting signals originated from membrane receptors and by soluble immunomodulators (23–25). In the particular case of lung cancer, tumor infiltrating NK cells are found in low numbers and display a dysfunctional phenotype characterized by impaired cytotoxic function, impaired degranulation, and decreased expression of activating receptors NKp30, NKp80, DNAM-1, CD16, and ILT2 (26–28). Moreover, as opposed to CD8^+^ T-cells, CD20^+^ B-cells, and DC-LAMP^+^ mature DCs, the prognostic value of NSCLC is apparently less linked to NK cell density and more depending on the phenotype of infiltrating NK cells (29, 30). Tumor-associated cells, including macrophages, myeloid-derived suppressor cells (MDSC), regulatory T cells (Treg), and/or CAFs contribute toward the characteristic immunosuppressive microenvironment in tumors, and may hinder the natural NK cell cytotoxic capacity (23, 31). Particularly, CAFs may inhibit NK cell-mediated killing of cancer cells, *via* expression of soluble mediators such as indoleamine-pyrrole 2,3-dioxygenase (IDO), matrix metalloproteinases, or prostaglandin E_2_ (PGE2) (32–34). These observations suggest that approaches that can interfere with the signaling between CAFs and NK cells may have therapeutic potential.

In the context of radiotherapy and cancer, few studies have explored NK cells responses to treatment. Radiation exposure has been shown to induce higher NK cell-mediated cytotoxicity of tumor cells *in vitro*, resulting from higher expression of NKG2D ligand on target cells (34). Additionally, studies with *in vivo* models indicate that RT may increase NK cell homing and cytotoxicity (35), or as shown in a recent study, adoptive transfer of *ex vivo* activated NK cells after irradiation can eliminate cancer stem-like cells and prolong survival compared with RT alone (36). Besides the observed direct effects, changes provoked by RT on tumor microenvironment (TME) elements that regulate NK cells phenotype and functions may indirectly affect NK cells anti-tumor activity. In this study, we explore if CAF-mediated immunoregulatory effects on NK cells are modified after exposure to different radiation regimens.

## Materials and Methods

### Human Material, Cancer-Associated Fibroblast Isolation, and Cultures

Human lung CAFs were isolated from freshly resected NSCLC tumor tissue taken from patients undergoing surgery at the University Hospital of Northern Norway (UNN), Tromsø, as previously described (9). Lung tumor specimens were randomly collected from eleven different patients (**Table 1**) and human blood (i.e., buffy coats) from six unrelated healthy donors were included in the study, under patient written informed consent. Briefly, NSCLC-derived CAFs were isolated based on mechanical mincing and enzymatic digestion of tissues by Accutase solution (Sigma-Aldrich, St. Louis, MO, USA; Cat. # A6964), followed by selective cell outgrowth in serum-supplemented medium. Established CAF cultures were characterized by the presence of lineage-specific markers; anti-human smooth muscle α-actin (α-SMA) (Abcam, Cambridge, UK; Cat. # ab7817, clone # 1A4) and anti-human fibroblast activation protein (FAP) (Vitatex, NY, USA; Cat. # MABS1001) (9). Isolated lung CAFs were cultivated in DMEM high glucose basal medium (Sigma Life Science, #D5796), supplemented with 10% FBS, 100 U/ml penicillin, and 100 μg/ml streptomycin and used for experimentation after the third and fourth passage (3–6 weeks old cultures), until passage seven. Normal skin fibroblasts (NFs) included in the study were purchased from Evercyte GmbH (Vienna, Austria; # fHDF/TERT166) and cultured in Gibco^®^ Opti-MEM™ reduced serum medium (Grand Island, US, # 31985-070) supplemented with 5% FBS, 100 U/ml penicillin, and 100 μg/ml streptomycin. Human lung cancer cell line A549 (lung adenocarcinoma) was purchased from LGC Standards AB (Borås, Sweden) and cultured in RPMI-1640 (Sigma Life Science, #R8758) containing 10% FBS supplemented with 100 U/ml penicillin and 100 μg/ml streptomycin. All methods involving human material were performed in accordance with relevant ethical guidelines and regulations. The Regional Ethical Committee of Northern Norway has approved the use of human material that has been included in this study (REK Nord 2014/401; 2016/714; 2016/2307).

**Table 1:** Cancer-associated fibroblast (CAF) donor information.

Donor #	Sex	Age	Tumor type	T-size (mm)	T-stage and N-stage
**1**	M	70	Squamous cell carcinoma	35	pT2aN0Mx
**2**	F	73	Adenocarcinoma	35	pT2aN0Mx
**3**	M	67	Squamous cell carcinoma	22	pT1cN0Mx
**4**	M	65	Squamous cell carcinoma	30	pT2aN0Mx
**5**	M	78	Adenocarcinoma	50	pT2bN0Mx
**6**	M	84	Adenocarcinoma	50	pT2bN0Mx
**7**	M	67	Adenocarcinoma	30	pT1cN1Mx
**8**	F	74	Adeno-squamous carcinoma	60	pT3N0Mx
**9**	M	65	Squamous cell carcinoma	24	pT1cN0Mx
**10**	M	81	Pleomorphic adenocarcinoma	46	pT2bN0Mx
**11**	F	59	Adenocarcinoma	21	pT1cN0Mx

### Irradiation of Cell Cultures

Adherent CAF cultures grown in T-75 flasks, six-well or 24-well culture plates were irradiated with high energy photons when cultures were 70–90% confluent, using a clinical Varian linear accelerator as previously described (9). Ionizing radiation was delivered to the cells either as a single high-dose (1x18 Gy) or in medium-dose fractionated schemes (3x6 Gy) at 24 h intervals. Standard parameters for dose delivery was depth 30 mm, beam quality 15 MV, dose-rate 6 Gy/min, and field size 20×20 cm. Cells were used for experimentation 3 to 5 days after irradiation for the (1x18 Gy) group and 1 to 3 days after the last radiation dose for the (3x6 Gy) group. In this paper, irradiated CAFs are referred as iCAFs.

### Isolation of Natural Killer Cells

**Figure 1:**
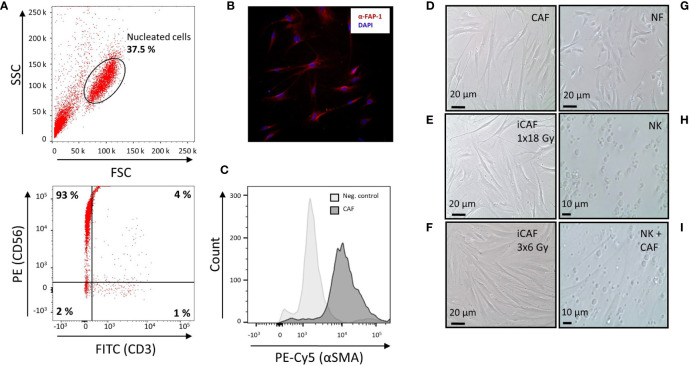
Natural killer (NK) cell purity, cancer-associated fibroblast (CAF) characterization, and radiation-induced morphological changes in CAFs. **(A)** Dot plots from flow cytometer analyses, illustrating 93% purity of NK cells defined as CD56^+^CD3^−^ upon negative selection by antibody-coated microbeads. **(B)** Immunostaining of cultivated CAFs, using CAF-specific FAP-1 antibody (red) and nuclear DAPI (blue). **(C)** Flow cytometry analyses of CAFs after immunostaining with CAF-specific αSMA antibody. Negative control is CAFs stained with isotype control antibody. **(D)** Representative culture of spindle-shaped, non-irradiated human lung CAFs at passage three. **(E)** Representative image of CAFs acquired 5 days after exposure to ionizing radiation (1x18 Gy). **(F)** Image of irradiated CAFs in culture, acquired 2 days after third dose of 6 Gy (3x6 Gy). **(G)** Culture of normal skin fibroblasts (NFs). **(H)** Image of non-adherent (CD56^+^CD3^−^) NK cells in monoculture. **(I)** Image of co-cultures consisting of adherent CAFs and non-adherent NK cells.

Peripheral blood mononuclear cells (PBMCs) were isolated from buffy coats using Lymphoprep-TM (StemCell Technologies, Vancouver, BC, Canada) and density-gradient centrifugations. Residual erythrocytes in the PBMC pool were removed by using red blood cells lysis buffer prior to negative selection of NK cells, based on the untouched separation through magnetic microbeads coated with specific antibodies against markers not expressed by NK cells (Miltenyi Biotec, # 130-092-657). By this method, CD56^+^CD3^−^ NK cells showed a purity above 90% from the fraction of mononuclear cells, as determined by flow cytometry (**Figure 1A**). Isolated NK cells were cultured in NK cell growth medium (RPMI-1640 with 10% FBS, 1% streptomycin/penicillin, 100 IU/ml IL-2, and 5 ng/ml IL-15) and kept in a humidified atmosphere (5% CO_2_, 37°C). Human IL-2 (# 130-097-748) and human IL-15 (# 130-095-765) were purchased from Miltenyi Biotec, Bergisch Gladbach, Germany.

### Co-Cultures and Natural Killer Cell Stimulation

Co-culture experiments were carried out with NK cells and fibroblasts in direct cell-cell contact. To this end, NK cells were isolated 48 h prior to co-culturing and incubated in NK cell growth medium in the presence of cytokines to promote cell activation and survival. Separately, monolayer cultures of irradiated CAFs (iCAFs), non-irradiated CAFs, or normal fibroblasts (NFs) were established in six-well (2x10^5^ cells/well) or 24-well plates (0.5x10^5^ cells/well). Fibroblast/NK cell cultures were established at ratio of (2:1) for experiments determining CAF cell surface receptors, NK cell cytotoxic activity and intracellular markers, and at a ratio (1:2) for experiments defining NK cell surface receptors and cytokine release. Upon initiation of co-cultures, the cells were incubated for additional 48 h (at 37°C) in NK cell growth medium. Thereafter, NK cells, fibroblasts, and supernatants were collected separately and used for further analyses.

### Natural Killer Cell Proliferation

Rates of NK cell proliferation were determined using the carboxy-fluorescein succinimidyl ester (CSFE) cell division assay kit (Cayman CHEMICAL, Michigan, USA, # 10009853). Briefly, freshly isolated NK cells were cultured overnight in NK cell growth medium, washed with prewarmed PBS, and then allowed to internalize CSFE in suspension for 15 min (at 37°C), as described in the kit. Next, NK cells were washed with culture medium, pellets of fluorescent cells were resuspended in NK cell growth medium and then established in either monocultures (1x10^5^ cells/well) or in co-cultures with CAFs or NFs (0.5x10^5^ cells/well) in 24-well plates. Rates of NK cell proliferation was determined after 1 and 5 days in co-culture by analyzing carboxyfluorescein succinimidyl ester (CFSE) fluorescence intensities on a BD FACS Aria III flow cytometer. Flow cytometric data were analyzed by FlowJo (TreeStar, OR, USA) software.

### Natural Killer Cell Cytotoxicity Assays

NK cell cytotoxic potential was determined by their capacity to kill K562 leukemic cells. Prior to initiating the assay procedure, fibroblasts and NK cells were co-cultured in NK cell growth medium at a ratio of 2:1 for 48 h at 37°C. Following co-culturing, NK cells (2.5×10^5^) were collected and further incubated with CFSE-labeled K562 non-adherent tumor cells (0.5×10^5^) (Sigma-Aldrich, # 89121407-1VL), effector to target ratio of 5:1, for 4 h at 37°C. Dead cells were identified by incorporation of propidium iodide (PI). To this end, PI staining solution (Miltenyi Biotec, # 130-093-233) was added to each sample just prior to analysis (final concentration 1 µg/ml). Data were obtained by flow cytometry from cells gated according to their scatter properties (FSC-A *vs.* SSC-A) and CFSE^+^. Percentage of PI-positive cells from the CSFE-positive K562-population was defined as killed target cells. NK cells treated with recombinant TGF-β (5 ng/ml, 48 h, PeproTech, USA, #100-21), cultured as monoculture, was used as negative control, whereas NK cells grown in co-culture with NFs represented the positive control in this experiment.

A similar cytotoxic assay was used to measure NK cell killing activity on irradiated CAFs. Twenty-four hours after the last dose of the fractionated radiation regime, and 3 days after single high dose-radiation exposure, CAF susceptibility for NK cell-mediated cell kill was determined in a cytotoxicity assay. After radiation exposure, CAFs were detached from the culture dishes, stained with CFSE and cultured at 0.5x10^5^ cells/well in suspension with/without NK cells (2.5x10^5^), effector-target ratio of 5:1, for 4 h at 37°C. Immediately before initiating flow cytometry analyses, cultures were stained with PI, to label dead and dying cells. Cells were gated according to their scatter properties (FCS-A *vs.* SSC-A) and CFSE^+^. Percentage of PI-positive cells from the CFSE-positive population was defined as dead target cells. To compensate for the spontaneous death of CAFs following growth in suspension cultures, CFSE-labeled CAFs were cultured alone for the same duration as the cytotoxicity assay and thereafter stained with PI (final concentration 1 µg/ml). The percentage of dead CAFs in monocultures was subtracted from the percentage of dead target cells from cultures with NK cells. NK cells cultured with CFSE-labeled K562 leukemic cells were used as a positive control for NK cell activity, whereas A549 was included as a negative control. Data from flow cytometry were analyzed using FlowJo (TreeStar, OR, USA) software.

### Quantitative Protein Determinations by ELISA

Concentrations of soluble cytokines and growth factors in samples were determined by enzyme-linked immunosorbent assay (ELISA). For these analyses, CAFs or NFs were co-cultured (48 h, 37°C) with NK cells in 24-well plates containing 0.5x10^5^ fibroblasts and 1x10^5^ NK cells per well (fibroblast-NK cell ratio 1:2), using the NK cell growth medium, as described above (§2.3). Human IFN-γ (R&D systems, Minneapolis, USA, # DY285B-05), human TNF-α (R&D systems, # DY210-05), human TGF-β (R&D systems, # DY240-05), and human PGE2 (Enzo, Switzerland, # ADI-900-001) were measured in culture supernatants using specific ELISA-kits and following the procedures described in each kit. Human IDO (Abcam, # ab245710) expression in CAFs was determined in cell lysates, as described in the specific ELISA-kit. Briefly, CAFs were co-cultured with NK cells in six-well plates (2.5x10^5^ CAFs and 5x10^5^ NK cells per well) for 48 h at 37°C in NK cell growth medium. Positive controls consisted of non-irradiated CAF cultures stimulated with IFN-γ (25 ng/ml) for 24 h before cell lysis, whereas negative controls consisted of non-treated CAFs. After elimination of non-adherent cells, CAFs were lysed directly in the wells by adding 200 µl cell extraction buffer (containing phosphatase inhibitor and aprotinin protease inhibitor) per well. Cell lysates were collected, incubated on ice for 15 min, spun down at 18.000 x g (20 min, 4°C) and the resulting cell extracts were diluted 100 times for further protein quantification by ELISA.

### Phenotypic Characterization of Cells by Flow Cytometry

Expression levels of activating and inhibitory NK cell receptors were determined by direct immuno-staining of selected surface proteins and analyzed by flow cytometry. Briefly, after co-culturing, (CD56^+^CD3^-^) NK cells (2.5×10^5^ cells/condition), were transferred to PBS-BSA buffer and stained with one of the following antibodies (Miltenyi Biotec): anti-NKG2A/CD159a (# 130-113-565, clone REA110), anti-NKG2D/CD314 (# 130-111-646, clone REA797), anti-NKp46/CD335 (# 130-112-119, clone REA808), anti-KIR3DL1/CD158e (# 130-099-693, clone DX9), anti-KIR2DL1/CD158a (# 130-119-138, clone REA284), anti-DNAM-1/CD226 (# 130-117-641, clone REA1040), and anti-LAMP1/CD107a (# 130-111-628, clone REA792). For intracellular staining, NK cells were initially preconditioned in co-cultures with CAFs, thereafter employed against K562 leukemic cells (for 4 h, 37°C), and next fixed and permeabilized using Inside Stain Kit (Miltenyi Biotec, # 130-090-477). Specimens were then stained with either anti-IFN-γ (#130-114-023, clone REA600), anti-TNF-α (# 130-120-063, clone REA656), anti-perforin (# 130-118-119, clone REA1061), or anti-granzyme B (# 130-116-486, clone REA226) dye-conjugated antibodies. Isotype controls consisted of REA control IgG1 (# 130-113-450) and isotype control IgG2a (#130-098-877) antibodies. Data were obtained by flow cytometry from cells gated according to their scatter properties (FSC-A *vs.* SSC-A) and doublet exclusion (FSC-A *vs.* FSC-H). Cell debris were excluded from the analyses based on scatter signals. Collected data from flow cytometry were analyzed by FlowJo (TreeStar, OR, USA) software.

**Figure 2:**
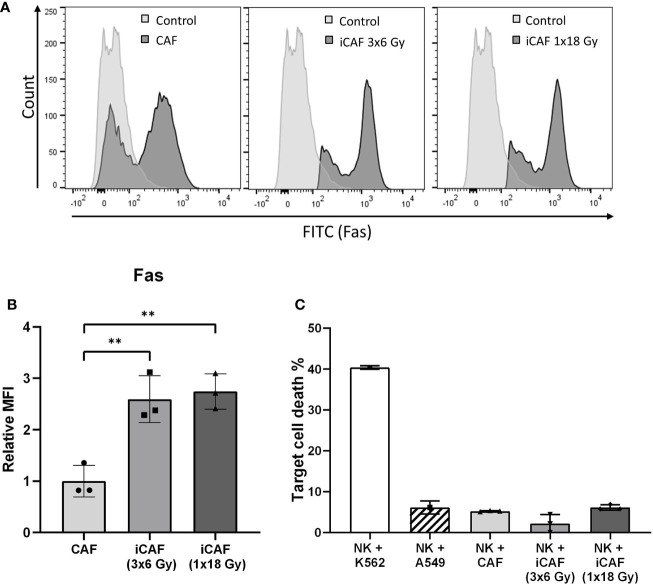
Radiation-induced Fas-receptor on cancer-associated fibroblasts (CAFs) and natural killer (NK) cell-mediated killing. Panels in **(A)** show flow cytometry histograms comparing surface expression of Fas receptor in irradiated and non-irradiated CAFs from a representative donor. Control histograms represent CAFs exposed to isotype control antibodies. **(B)** Quantitative expression of Fas receptor presented as median fluorescence intensity (MFI) calculated from three independent CAF donors. **(C)** NK cell cytotoxicity assay measuring NK cell mediated cell killing of irradiated or non-irradiated CAFs as targets, in addition to two different control target cell types at NK to target ratio 5:1. Bars represent mean (± SD) from three independent CAF donors. Statistics and *P*-values between NF and CAFs were determined using one-way ANOVA with Tukey correction for multiple comparisons. ** indicates *P* < 0.01.

Expression of checkpoint receptors on CAFs upon radiation exposure was examined by direct immunostaining of selected surface proteins. Briefly, 4–5 days after irradiation, CAFs were transferred to PBS-BSA buffer and stained with one of the following dye-conjugated anti-human antibodies (Miltenyi Biotec): PD-L1 (# 130-122-815, clone REA1197), CD155/PVR (# 130-119-176, clone REA1081), anti-HLA-E/MHC-I (# 130-117-549, clone REA1031), anti-CD112 (# 130-122-782, clone REA1195), and anti-Fas/CD95 (# 130-113-068, clone REA738). Data were obtained by flow cytometry from cells gated according to their scatter properties (FSC-A *vs.* SSC-A), doublets exclusion (FSC-A *vs.* FSC-H). For measurements of Fap expression related to CAF cytotoxicity (**Figure 2C**), cell debris and dead cells were excluded from the analysis based on scatter signals and PI fluorescence, as cells exhibiting PI signal are excluded from viable cells. Collected data from flow cytometry were analyzed by FlowJo (TreeStar, OR, USA) software.

### Statistical Analyses

All statistical analyses were performed using GraphPad Prism (GraphPad Software, Inc., La Jolla, CA). Comparison of data between three or more experimental groups were analyzed using one- or two-way ANOVA followed by either Tukey or Dunnett *post hoc* corrections for multiple comparisons. Level of significance was defined as *P*<0.05. Results were presented in graphs, where each CAF donor was plotted as an individual dot in the dataset. In figures generated from ELISA analyses, only read-outs within the detection limit of the assay are presented.

## Results

### Ionizing Radiation-Induced Morphological Changes in Cultured Cancer-Associated Fibroblasts

Considering that different radiation schemes may trigger distinct biological effects, in our study we have included two experimental groups of irradiated CAFs; one exposed to a fractionated regimen (3x6 Gy) and the other to single high-dose radiation (1x18 Gy). The chosen radiation doses and schemes are in line with RT regimens given in the clinics to lung cancer patients in the context of SBRT. Initially, the purity of isolated NK cells was defined by determining the fraction of CD56-positive and CD3-negative (CD56^+^CD3^−^) mononuclear cells by flow cytometry. Representative flow cytometry dot plots revealed a high degree of purity, with a total of 93% of isolated NK cells demonstrating a CD56^+^CD3^−^-phenotype (**Figure 1A**).

Isolated NSCLC-derived CAFs were identified by their expression of the lineage specific markers FAP-1 (**Figure 1B**) and α-SMA (**Figure 1C**).

Upon radiation exposure, the various CAF cultures were morphologically examined by phase-contrast microscopy. Cultures of non-irradiated CAFs appeared with flattened, elongated, and spindle-shaped morphology, characteristic of tumor fibroblasts (**Figure 1D**), whereas irradiated CAFs (1x18 Gy) demonstrated enlarged and extensively flat morphology (**Figure 1E**). Similar flattened morphology was exhibited by CAFs irradiated at 3x6 Gy (**Figure 1F**), although the rates of cell senescence were slightly lower than for the 1x18 Gy group [data not shown (9)]. In contrast to the flattened and elongated morphology of both normal fibroblasts (NF) (**Figure 1G**) and CAFs (**Figures 1D–F**), NK cells are by nature non-adherent, much smaller than CAFs, and with a ball-shaped morphology (**Figure 1H**). To explore CAF-mediated immuno-regulatory effects on NK-cells, the two cell types were cultured together, allowing both direct cell-to-cell interactions and paracrine signaling. A representative co-culture of adherent fibroblasts (CAFs) and non-adherent NK-cells is also shown (**Figure 1I**).

### Irradiated Cancer-Associated Fibroblasts Are Not Killed by Natural Killer Cells, Despite the Upregulation of Fas Receptor

NK cells are known to eliminate stressed or damaged cells, and can supposedly perform cell killing through the exposure of death receptor ligands, including Fas ligand (FasL) and TNF related apoptosis-inducing ligand (TRAIL). We checked if radiation-induced cell damage could trigger expression of death receptors (Fas) on radioresistant CAFs, possibly turning iCAFs into targets of NK cell-mediated killing. Notably, Fas was robustly upregulated (2.5-fold) on the two iCAF groups (**Figures 2A, B**). However, NK cell-mediated CAF killing, as defined by uptake of PI, was not enhanced in iCAFs compared to control CAFs, and remained with very low rates (<10%) in all experimental groups (**Figure 2C**).

### Cancer-Associated Fibroblasts Sustain Their Capacity to Suppress Natural Killer Cell Proliferation After Radiation

**Figure 3:**
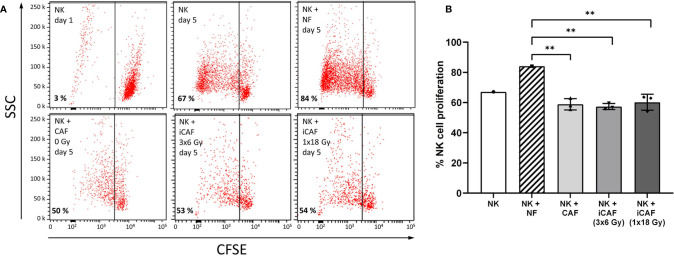
Effects of cancer-associated fibroblasts (CAFs) on natural killer (NK) cell proliferation. **(A)** Representative flow cytometry dot plots of the different experimental groups, showing percentage of carboxy-fluorescein succinimidyl ester (CSFE)-labeled NK cells on a side-scatter axis. **(B)** Bars representing mean (± SD) values for NK cell proliferation obtained from experiments with three different CAF donors. NK cells/NFs co-cultures were used as positive control, and the three experimental groups of NK cells/CAFs co-cultures were compared with the normal fibroblast (NF) group. Statistical *P*-values between NFs and CAFs co-cultures were determined using one-way ANOVA with Tukey correction for multiple comparisons. ** indicates *P* < 0.01.

As occurring with other lymphoid cells, the activation state of NK cells correlates with their proliferation rates. In this study, NK cell proliferation was determined in CSFE (fluorescence) dilution assays, with NK cells grown in the presence of irradiated or non-irradiated CAFs. In all experimental groups, the inflammatory cytokines IL-2 and IL-15 were present in the NK cell growth medium to support NK cell activation and survival. Despite the ubiquitous presence of cytokines, NK cells were much more proliferative in co-cultures with normal fibroblasts (NFs) than in monocultures (**Figure 3**). However, a considerable reduction in cell proliferation (about 37%) was observed for NK cells co-cultured with irradiated or non-irradiated CAFs compared to NFs (*P<0.01*) (**Figure 3B**). These results demonstrate the inhibitory potential of CAFs over NK cells and show that this effect is sustained after radiation.

### Reduced Natural Killer Cell Cytotoxicity After Co-Culturing With Irradiated and Non-Irradiated Cancer-Associated Fibroblasts

**Figure 4:**
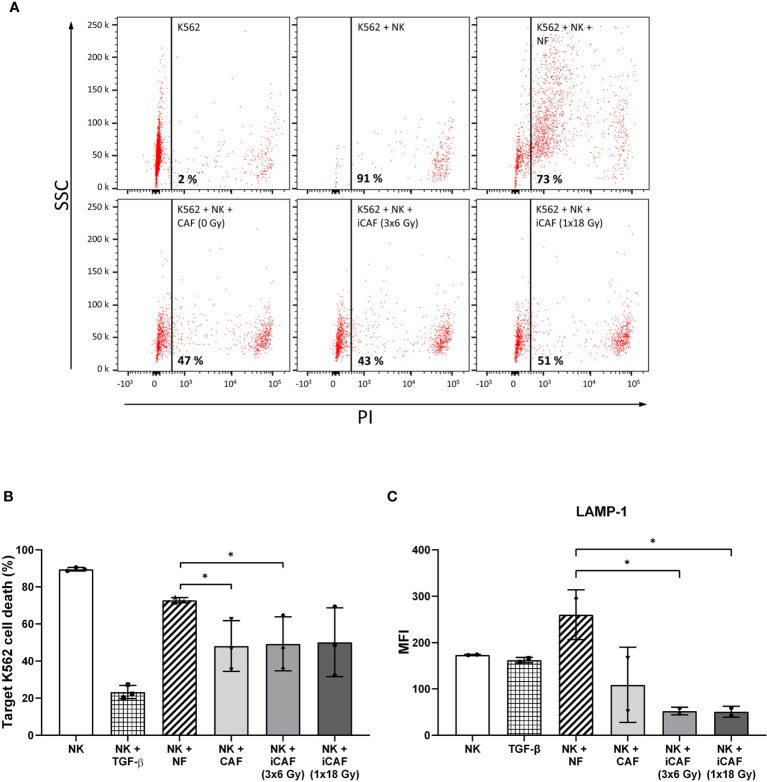
Effects of cancer-associated fibroblasts (CAFs) on natural killer (NK) cell cytotoxic activity. Cytotoxic activity of NK cells co-cultured with fibroblasts was analyzed against CSFE-labeled K562 leukemic tumor cells at NK to target ratio of 5:1. Results were evaluated by flow cytometry and presented as percentage propidium-iodide positive (dead) cells *versus* side scatter activity (SSC). TGF-β treated NK cells served as control for repressed cytotoxic activity. Panels in **(A)** represent flow cytometry dot-plots from a representative experiment with one of the CAF donors. In **(B)**, NK cell cytotoxic activity is represented as mean (± SD) values from flow experiments with three different CAF donors. Statistical *P*-values between mixed cultures with NF and CAFs were determined using one-way ANOVA with Tukey correction for multiple comparisons. Similarly, in **(C)**, NK cell degranulation values are calculated as levels of LAMP-1 (CD107a) present on the NK cell surface after being employed against K562 leukemic tumor cells (for 4 h), at a NK to target ratio of 5:1, and an initial co-culturing with CAFs. Data represent mean (± SD) values from three different donors. * indicates *P* < 0.01.

NK cells are innate lymphoid cells with an intrinsic selectivity and capacity to kill cancer cells over normal healthy cells without the requirement for prior sensitization. The influence of CAFs on the killing potential of NK cells was determined by using a cytotoxicity assay against the leukemic cancer cell line K562. NK cells were pre-conditioned with either NFs, irradiated or non-irradiated CAFs in co-cultures for 48 h. Upon co-culturing, NK cells were collected and used directly in cytotoxicity assays, using (CSFE-labeled) K562 tumor cells as targets and PI incorporation as indicator of cell death. Results indicate that NK cells in monocultures exert maximum killing activity toward tumor cells, whereas this activity was strongly blocked in the presence of TGF-β (negative control) and slightly reduced in the NK/NF co-culture group (**Figures 4A, B**). Importantly, NK cells co-cultured with CAFs (irradiated and non-irradiated) demonstrated significantly reduced killing capacity (*P<0.05*) compared to the NF group (**Figure 4B**).

Cytotoxic actions by NK cells are mediated by exocytosis of perforin-containing secretory lysosomes (lytic granules). To gain quantitative information on the extent of lytic granules release from NK cells, we performed a degranulation assay, which essentially consists of quantifying the presence of lysosome-associated membrane protein-1 (LAMP-1, also called CD107a) on the surface of NK cells. For this assay, NK cells were preconditioned under different co-culture conditions and thereafter employed against K562 leukemic target cells. Our results indicate that NK cells cultured in the presence of CAFs display reduced degranulation rates as compared to NK/NF co-cultures, and that irradiated CAFs exert a further enhanced reduction to about 80% (*P*<0.05) (**Figure 4C**).

Collectively, these results indicate that the resulting reduced rates of NK cell-mediated tumor cell killing in the presence of CAFs correlates with the extent of NK cell degranulation.

### Cancer-Associated Fibroblast-Mediated Effects on Natural Killer Cell Activation Markers

**Figure 5:**
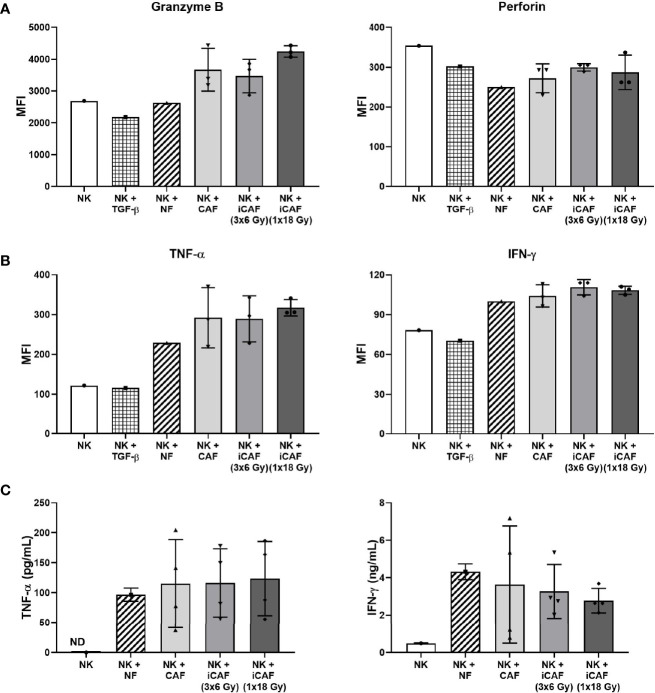
Effects of cancer-associated fibroblasts (CAFs) on natural killer (NK) cell activation markers. NK cells were incubated in mixed cultures with NFs or CAFs. NK cells treated with TGF-β served as control of repressed functional activity. Following co-culturing, NK cells were analyzed for **(A)** intracellular expression of cytotoxic enzymes perforin and granzyme B after being employed against K562 cells (NK to K562 ratio 5:1); **(B)** intracellular expression of pro-inflammatory cytokines IFN-γ and TNF-α after exposure to K562 cells (5:1); and **(C)** released levels of IFN-γ and TNF-α in culture media (fibroblast to NK ratio 1:2). Results in **(A, B)** were measured by flow cytometry, whereas cytokine release in **(C)** was quantified by ELISA. For intracellular markers, signal intensities are expressed as median fluorescence intensity (MFI). Data represents mean (± SD) values from three different CAF donors. Statistical *P*-values between NF and CAFs were determined using one-way ANOVA with Tukey correction for multiple comparisons. Extracellular levels in **(C)** are presented as mean (± SD) values from four random CAF donors.

We next sought to analyze intracellular levels of pro-inflammatory cytokines and lytic enzymes associated with NK cell activation and effector functions. For these experiments, NK cells were grown in co-cultures with fibroblasts as before, then collected, and employed against the tumor cell line K562 (4 h, 37°C), followed by fixation, permeabilization, and staining with antibody-markers against cytokines IFN-γ, TNF-α, and cytolytic enzymes perforin and granzyme B. Our results indicate that intracellular levels of granzyme B were slightly elevated in NK cells grown in the presence of iCAFs/CAFs rather than NFs, but the differences were not significant (**Figure 5A**). The levels of perforin were slightly reduced in all co-culture conditions compared to NK cell monocultures, and no differences were observed between NF and CAFs groups or between irradiated and non-irradiated CAFs. Moreover, intracellular levels of the proinflammatory cytokines TNF-α and IFN-γ were considerably enhanced when comparing NK cells monocultures with the co-culture groups (**Figure 5B**), however, differences for both TNF-α and IFN-γ are marginal when comparing the NF-group with CAF/iCAF groups, and do not reach significance.

As an additional approach to evaluate NK cell activation, we also checked for release of TNF-α and IFN-γ into the incubation medium during co-culturing with CAFs or NFs. Of note, secretion of TNF-α or IFN-γ by CAFs was non-detectable in monoculture CAF supernatants (not shown), indicating that the secreted cytokines were coming essentially from NK cells. Our results indicate that NK cells secrete considerably higher levels of cytokines during co-culture conditions than in monocultures (**Figure 5C**). However, no significant differences were observed for TNF-α or IFN-γ secretion between the NF group and the other CAFs groups, or, when comparing irradiated and non-irradiated CAFs groups.

### Irradiation Does Not Alter Cancer-Associated Fibroblasts Release of Immunomodulators

**Figure 6:**
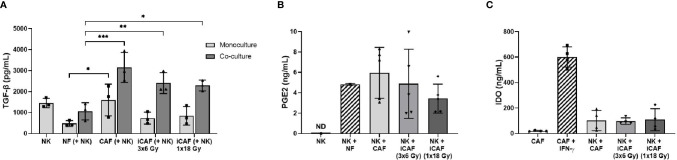
Effects of radiation on cancer-associated fibroblast (CAF)-derived immunomodulators. **(A, B)** Protein levels of secreted TGF-β and PGE2 in culture supernatants measured by ELISA. **(C)** Intracellular IDO protein expression in CAF cell lysates analyzed by ELISA. In **(C)**, positive control is non-irradiated CAFs stimulated with IFN-γ and negative control non-irradiated CAFs cultured without stimulation. Data represents mean (± SD) values from three **(A)**, five **(B)** or four **(C)** different CAF donors. *P*-values between NFs and CAFs in **(A, B)** were determined using one-way ANOVA with Tukey correction for multiple comparisons, whereas *P*-values between groups of monocultures and co-cultures in **(C)** were determined using two-way ANOVA with Dunnett correction for multiple comparisons. *P < 0.05, **P < 0.01 and ***P < 0.001.

Many of the described effects exerted by CAFs on immune cells are mediated *via* secretion of soluble signal molecules with immunosuppressive potential. To determine if radiation could have direct effects on the expression of immunosuppressive factors by CAFs, we evaluated the secretion of three relevant immunomodulators during co-culturing, namely TGF-β, PGE2, and IDO. We found that both NK cells and CAFs secrete considerable amounts of TGF-β, indicating that measured TGF-β in co-cultures come from both cell sources (**Figure 6A**). Importantly, TGF-β levels in supernatants of NK/CAF co-cultures were significantly (two-fold) increased (97%, *P*=0.002) compared to NK/NF co-cultures. In co-cultures with irradiated CAFs (NK/iCAF), TGF-β levels were slightly reduced compared to control CAFs, but significantly higher than in NK/NF co-cultures (*P*<0.05).

In contrast, PGE2 was undetectable in NK cells monocultures (as expected) but expressed in both NK/NF and NK/CAF co-cultures. PGE2 levels were slightly increased in control CAFs supernatants compared to NFs without reaching significance. Radiation seemed to curtail PGE2 expression to some extent compared to non-irradiated CAFs group, although the differences were not significant due to large inter-donor variations (**Figure 6B**). IDO expression by CAFs was strongly induced after exogenous administration of IFN-γ, however, expression of IDO was kept at very low levels during co-cultures and no differences between CAFs and iCAFs groups were observed (**Figure 6C**).

### Irradiation Does Not Alter Cancer-Associated Fibroblast-Mediated Regulation of Natural Killer Cell Receptors

**Figure 7:**
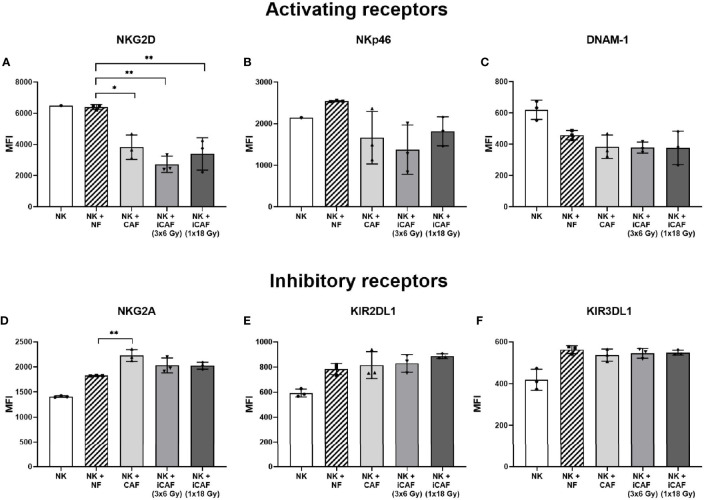
Effects of cancer-associated fibroblasts (CAFs) on natural killer (NK) cell surface receptor expression. Following co-culturing with fibroblasts, isolated NK cells were analyzed by flow cytometry for their expression of NK cell surface markers **(A)** NKG2D, **(B)** NKp46, **(C)** DNAM-1, **(D)** NKG2A, **(E)** KIR2DL1 and **(F)** KIR3DL1. Results are presented as median fluorescence intensity (MFI) and data represents mean (± SD) values from experiments with three different CAF donors. Statistical *P*-values between NK/NFs and NK/CAF-groups were determined using one-way ANOVA with Tukey correction for multiple comparisons * and ** indicates *P* < 0.05 and *P* < 0.01, respectively.

Effector functions in NK cells are governed by signals transmitted through multiple receptor–ligand interactions. To investigate whether radiation alters the capacity of CAFs to modulate activation signaling pathways in NK cells, we checked expression levels of an array of cell surface receptors in NK cells after co-culturing with normal fibroblasts, iCAFs or non-irradiated CAFs. The panel of studied receptors comprised both activating receptors, also referred to as NKARs in this study, (NKG2D, NKp46, and DNAM-1), as well as inhibitory receptors or NKIRs (NKG2A, KIR2DL1, and KIR3DL1). Our results indicate that the presence of CAFs induces a significant reduction (40–50%) in surface expression of the stimulatory receptor NKGD2, compared to co-cultures with NFs (*P<0.01*) (**Figure 7A**). Importantly, similar outcomes were observed after co-culturing NK cells with either one of the two iCAF groups. A tendency for lower expression of NKp46 and DNAM-1 was observed in CAFs co-culture groups compared to NFs group without reaching significance (**Figures 7B, C**). Regarding inhibitory receptors, we observed a significant (*P*<0.01) augmentation in expression levels of NKG2A in the non-irradiated CAF group (**Figure 7D**) compared to the NF group. In contrast, expression levels of KIR2DL1 and KIR3DL1 remain unchanged in all co-culture experimental groups (**Figures 7E, F**).

### Changed Expression Levels of Natural Killer Receptor Ligands on Irradiated Cancer-Associated Fibroblasts

**Figure 8:**
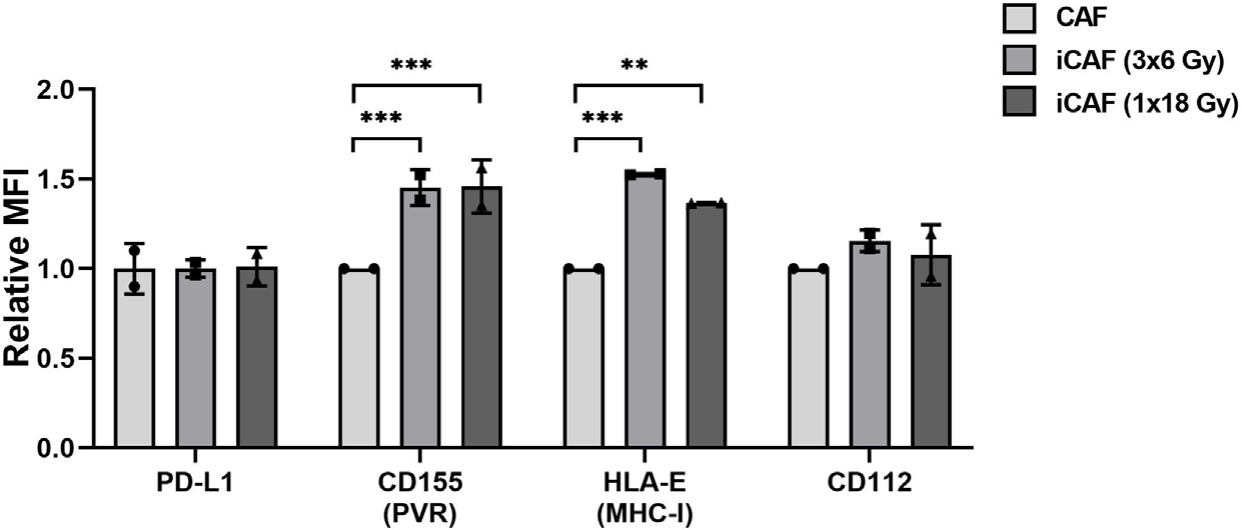
Effects of radiation on cancer-associated fibroblast (CAF) surface expression of checkpoint molecules. Surface expression of checkpoint ligands PD-L1, CD155, CD112, and HLA-E (MHC-I) were analyzed by flow cytometry on control and irradiated CAFs (iCAFs). Results are expressed as fold non-irradiated CAF median fluorescence intensity (MFI). Bars represent mean (± SD) values from two different CAF donors. Statistical *P*-values between control CAFs and irradiated CAF-groups were determined using two-way ANOVA with Dunnett correction for multiple comparisons. **P < 0.01 and ***P < 0.001.

Inhibitory signals for NK cells can also be mediated *via* direct cell-cell interactions and engagement of inhibitory checkpoint receptors on the cell surface. Well-described checkpoint receptors in NK cells comprise molecules such as inhibitory programmed cell death protein-1 (PD-1), NKG2A, and killer-cell immunoglobulin-like receptors (KIRs). In our study, we have examined whether radiation is able to alter the CAF surface expression of inhibitory ligands to these receptors, including PD-L1, PVR (CD155), nectin-2 (CD112), and HLA-E (MHC-I). Results from this experiment demonstrate that surface levels of checkpoint molecules PD-L1 and CD112 are expressed at similar levels on irradiated and non-irradiated CAFs (**Figure 8**). However, both the poliovirus receptor (PVR/CD155) and the “self-receptor” HLA-E/MHC-I demonstrated substantial donor-independent upregulation (≈1.5-fold, *P<0.01*) in the two irradiated-CAF groups.

## Discussion

During the course of radiotherapy, radiation-induced changes on the tumor microenvironment may, directly or indirectly, affect NK cell anti-tumor functions. In this study, we have explored if and how CAF-mediated immunoregulation against NK cells is changed after ionizing radiation exposure. We have observed that NK cells in direct contact with CAFs from NSCLC tumors display a molecular profile characteristic of tolerogenic NK cells, indicated by **i)** reduced proliferation rates and cytotoxic capacity; **ii)** reduced surface expression of LAMP-1 (CD107a) and triggering receptors (NKG2D, NKp46, DNAM-1); **iii)** enhanced surface expression of some inhibitory receptors (NKG2A) (**Table 2**). Of note, ionizing radiation, delivered as single high- or fractionated medium-dose to CAFs, triggered elevated surface expression of Fas death receptor on CAFs, but this response was insufficient to activate NK cell-mediated immune recognition and elimination. In addition, radiation exposure did neither improve nor worsen the general CAF-mediated immunosuppression on NK cell function. However, analyses of selected NK receptor ligands on irradiated CAFs revealed prominent upregulation of surface HLA-E and PVR/CD155, that are ligands for NK inhibitory receptors NKG2A and TIGIT/CD96, and activating receptor DNAM-1, respectively.

**Table 2:** Summary of main findings.

NK cells co-cultivated with		NF	A549	CAF 0 Gy	iCAF 3x6 Gy	iCAF 1x18Gy	Effects from IR	Fold upreg
Functional assays	Function							
Proliferation	Proliferation	Ctrl	_	↓	↓	↓	no	_
Cytotoxic on K562 cells	Ability to kill K562 cells	Ctrl	_	↓	↓	↓	no	_
Cytotoxic on CAFs	Ability to kill CAFs	_	Ctrl	↔	↔	↔	no	_
**NK cell surface receptors**				
NKG2D	NKAR	Ctrl	−	↓	↓	↓	no	–
DNAM1	NKAR	Ctrl	−	↔	↔	↔	no	−
NKp46	NKAR	Ctrl	−	↓	↓	↓	no	−
NKG2A	NKIR	Ctrl	−	↑	↔	↔	no	–
KIR2DL1	NKIR	Ctrl	−	↔	↔	↔	no	−
KIR3DL1	NKIR	Ctrl	−	↔	↔	↔	no	−
LAMP-1	Degranulation	Ctrl	−	↓	↓	↓	no	–
**NK cell i.c. markers**								
IFN-γ	Immunostimulant	Ctrl	−	↔	↔	↔	no	−
TNF-α	Immunostimulant	Ctrl	−	↔	↔	↔	no	−
Granzyme-B	Apoptosis	Ctrl	−	↑	↑	↑	no	−
Perforin	Pore formation	Ctrl	−	↔	↔	↔	no	−
**Secreted factors**								
TNF-α	Immunostimulant	Ctrl	−	↔	↔	↔	no	−
IFN-γ	Immunostimulant	Ctrl	−	↔	↔	↔	no	−
TGF-β	Immunosuppression	Ctrl	−	↑	↑	↑	no	−
PGE2	Immunosuppression	Ctrl	−	↔	↔	↔	no	−
IDO	Immunosuppression	−	−	Ctrl	↔	↔	no	−
**CAF receptors**								
PD-L1	Immunosuppression	−	−	Ctrl	↔	↔	no	−
Nectin-2/CD112	NK cell ligand	−	−	Ctrl	↔	↔	no	−
PVR/CD155	NK cell ligand	−	−	Ctrl	↑	↑	yes	1.5
HLA-E	NK cell ligand	−	−	Ctrl	↑	↑	yes	1.5
Fas/CD95	NK cell death receptor	–	−	Ctrl	↑	↑	yes	2.6

NK cells are major contributors to the innate immune defenses, with the ability to recognize and eliminate damaged cells, virus infected cells or (pre)malignant cells, thus performing crucial immune surveillance of the host. In our study, we first checked if irradiated CAFs could be recognized and killed by cytokine-activated NK cells. We and others have previously demonstrated that CAFs are extremely resistant to IR (9, 10), surviving to high-doses of radiation, but becoming prematurely senescent, with reduced proliferation, migration and invasion capacity, and displaying permanent DNA damage responses (9). In this study, we show that exposure to radiation also triggers Fas receptor surface expression on CAFs, however, this phenomenon turns out insufficient to initiate immune recognition and cytolytic actions by NK cells. Resistance to Fas-mediated apoptosis has also been reported in (normal) human lung fibroblasts (37), confirming the idea of fibroblasts as a highly robust cell type (7). Fas (CD95) and Fas ligand (CD95L) is traditionally considered as a death receptor-ligand system that triggers apoptosis to maintain immune homeostasis. However, recent data indicate that CD95 engagement may also trigger non-apoptotic signals that promote inflammation and tumor progression (38–41). A recent study by Pereira et al. proposes that increased HLA-E expression by senescent cells contributes to the evasion of NK cell-mediated immune clearance (42). Accordingly, in our study we also observe upregulation of HLA-E on irradiated CAFs, which could represent one of the counteracting mechanisms to repress NK cell killing signals.

NK cells are equipped to exert powerful cytotoxic activity against malignant cells, however, immuno-subversion by stromal components of the tumor microenvironment play a major role in preventing NK cell responses against tumors. The role of CAFs as suppressors of NK cell anti-tumor actions has been demonstrated in different cancer types (32, 33, 43). In this study, we demonstrate that CAFs from NSCLC also exert immunosuppressive effects against cytokine-activated NK cells. Both the proliferation rates and the cytolytic potential of NK cells became attenuated in the presence of CAFs, as compared to co-cultures with normal skin fibroblasts. In accordance with the findings of CAF-mediated reduction in NK cell cytotoxicity, we found that surface levels of lysosomal-associated membrane protein-1 (LAMP-1)/CD107—a marker of lytic granule exocytosis—were reduced in all experimental groups that included CAFs. Interestingly, irradiated CAFs, as opposed to control CAFs, appeared to maximally block the surface appearance of LAMP-1.

In contrast, intracellular levels of cytolytic proteins granzyme B and perforin remained unchanged, or even slightly enhanced, in all CAF-treated (co-culture) groups. Similar to us, other groups have reported reduced cytolytic activity in connection to reduced levels of activating NKG2D, without changes in perforin content (26). Hazeldine et al. (44) explored mechanisms for reduced NK cell cytotoxicity in the context of physiological aging and found—like us—that intracellular levels of perforin and granzyme B was similar for NK cells whether isolated from old or young donors. Collectively, several studies have indicated that the intracellular levels of cytolytic enzymes do not necessarily correlate with the cytotoxic rates exerted by NK cells.

The ignition of cytotoxic activity in NK cells is mainly regulated by the interplay between inhibitory and activating signals originating at the plasma membrane of NK cells from NKIRs and NKARs, respectively (22, 23, 25). In our study, we have checked the expression of major NKIRs and NKARs on NK cells upon co-culturing with irradiated and control CAFs. In agreement with previous studies performed in different cancer models (32, 33, 43), we observe a significant reduction in the expression of NKG2D and some (non-significant) reduction in expression of NKp46 and DNAM-1 on CAF-educated NK cells. Additionally, we observe a significant upregulated expression of the inhibitory receptor NKG2A. Both irradiated and non-irradiated CAFs exert comparable effects. These findings suggest that the presence of CAFs, whether irradiated or not, is able to skew NK cells toward a tolerogenic phenotype, therefore contributing to NK cells immunosuppression.

To complete the analyses of activating and inhibitory signaling, we have also studied the expression of NKIR and NKAR ligands on irradiated and control CAFs. One major finding of the present study is that radiation-induced senescent CAFs displayed upregulated surface amounts of the non-classical MHC-I molecule HLA-E, compared to non-irradiated CAFs (˜50%). Intriguingly, the corresponding inhibitory checkpoint receptor, NKG2A, was also slightly upregulated upon co-culturing with any of the three CAF-groups. As demonstrated by others (42), the NKG2A/HLA-E axis could play a central role in evasion of immune clearance of senescent cells, and thus may represent a main mechanism behind immune escape of irradiated CAFs. Another major finding of the study is a prominent upregulation of the poliovirus entry receptor (PVR/CD155) on irradiated CAFs. PVR is frequently overexpressed in human malignancies, and is associated with tumor progression, poor prognosis and immune escape (45, 46). Through its interaction with the NKIRs TIGIT and CD96 and the NKAR DNAM1, PVR is involved in the immunoregulation of NK cell responses (22). Recent reports indicate that the group of DNAM1/NKG2D ligands on target cells, including PVR and Nectin2/CD112, are upregulated in response to cellular stress, like DNA damage responses induced by chemotherapy, ionizing radiation, and viruses (47). Our findings on PVR upregulation on irradiated CAFs are therefore in line with observations by others. In contrast, both CD112/Nectin-2 and PD-L1 displayed similar surface levels on all CAF-groups, irrespective of radiation exposure. Nevertheless, despite the observed prominent enhancement of inhibitory ligands HLA-E and PVR upon irradiation, we could not see differences between irradiated and non-irradiated CAFs on NK cells functional assays, including proliferation rates, cytokine release, or killing activity. HLA-E exert primarily inhibitory effects on NK cells *via* interaction with the corresponding inhibitory receptor NKG2A. On the other hand, CD155 (PVR) exert activating signals on NK cells *via* its interaction with DNAM1 receptor. It is plausible that the sum of stimulatory and inhibitory signals leads to a neutralization of the net effects, and therefore no functional differences are observed.

In addition to cell-contact mediated signaling, NK cell phenotype and functions can also be regulated by soluble immunomodulators. Earlier studies using CAF/NK cell co-culture systems propose a prominent role played by CAF-derived PGE2 and IDO in mediating NK cells immunosuppression (32, 43, 48). Guided by such studies, we have compared the expression PGE2 and IDO in irradiated and control CAFs during co-culturing. Our data show that PGE2 is readily secreted into supernatants of CAFs and NF co-cultures, slightly decreased in the group of (1x18 Gy) iCAFs but without reaching significance. On the other hand, IDO is highly expressed in IFN-γ-treated CAFs but expressed at very low levels in both irradiated and control CAFs. These results, showing no differences in expression of major NK cells immunomodulators by CAFs after radiation exposure, harmonize with the outcomes in functional assays, where no differences are observed between irradiated and non-irradiated CAF-groups. Moreover, we also examined expression of TGF-β, as this growth factor is considered a major suppressive factor of NK cell functions (49, 50), and powerfully counteract anti-tumor immune responses from radiotherapy (51) and immunotherapy (52). We found elevated levels of TGF-β in all (NK/CAF) co-culture supernatants, with the most prominent upregulated quantities (97%, two-fold) induced by non-irradiated CAFs. In contrast, co-cultures with irradiated CAFs demonstrated a small (but non-significant) reduction in TGF-β levels, that on average was still significantly higher than average levels in NK/NF co-cultures. In previous studies, we have quantified secreted levels of TGF-β from non-irradiated/irradiated CAF monocultures and found as well minor impact of radiation in terms of TGF-β release from CAFs (13). It is plausible that some inhibitory effects exerted by CAFs in our system are mediated by TGF-β, and that similar levels of secreted TGF-β by irradiated and non-irradiated cells translates also into similar inhibitory effects on NK cells.

The direct impact of RT on NK cell anti-tumor activity in general has been scarcely investigated. Earlier reports suggest that RT may affect NK cell biology and functions, both directly and indirectly. Falcke et al. (53) reported that NK cells display a rather radiosensitive phenotype compared to monocytes and other myeloid cells, with radiation doses above >1 Gy, as used for malignancies, causing cell apoptosis (53), impaired cytotoxic, and activation capacity (54). However, others have reported NK cell functions to become abrogated upon a single dose of 30 Gy (54). In contrast, low-dose radiation in the mGy range may exert beneficial anti-tumor effects, as it apparently induces NK cell proliferation and secretion of effector proteins (55, 56). Indirect effects triggered by RT on NK cell functions comprise effects on immune cells with immunoregulatory functions such as macrophages, myeloid-derived suppressor cells, regulatory T cells and DCs, and changes in tumor cell exhibited ligands directly linked to cancer cell immune recognition (34, 57, 58). In our study, we focus on CAFs, a frequently forgotten cell type of the TME with powerful immune regulatory properties. Our data indicate that irradiated CAFs escape immune recognition and retain their immunosuppressive effects over NK cells, suggesting that radiation do not alter substantially the preexisting crosstalk between CAFs and NK cells in the TME.

## Data Availability Statement

The raw data supporting the conclusions of this article will be made available by the authors, without undue reservation.

## Author Contributions

Data collection: NY, KL, RB, AI, TH. Evaluation and interpretation of results: NY, KL, IM-Z, TH. Writing, reviewing the manuscript: NY, KL, IM-Z, TH. Concept and design: IM-Z and TH. All authors contributed to the article and approved the submitted version.

## Funding

This work was supported by the Norwegian Regional Health Authorities, grants # SFP1137-13, SPF1138-13, HNF1423-18, and HNF1373-17; The Norwegian Cancer Society and the Aakre Foundation. The publication charges for this article have been funded by a grant from the publication fund of UiT The Arctic University of Norway.

## Conflict of Interest

The authors declare that the research was conducted in the absence of any commercial or financial relationships that could be construed as a potential conflict of interest.

## Abbreviations

CAFs, cancer-associated fibroblasts; iCAFs, irradiated cancer-associated fibroblasts; Gy, Gray; IR, ionizing radiation; NKAR, NK cell activating receptor; NKIR, NK cell inhibitory receptor; NSCLC, non-small cell lung cancer; PBMC, peripheral blood mononuclear cells; UNN, University Hospital of Northern Norway.
