# Supplementary material for: Irradiated Tumor Fibroblasts Avoid Immune Recognition and Retain Immunosuppressive Functions Over Natural Killer Cells
Source: Front Immunol. 2021 Jan 22;11:602530. doi: 10.3389/fimmu.2020.602530 (PMC7874190; doi:10.3389/fimmu.2020.602530)
Supplement: Supplementary file 1 [file DataSheet_1.docx]

Supplementary Material

# Supplementary Figures


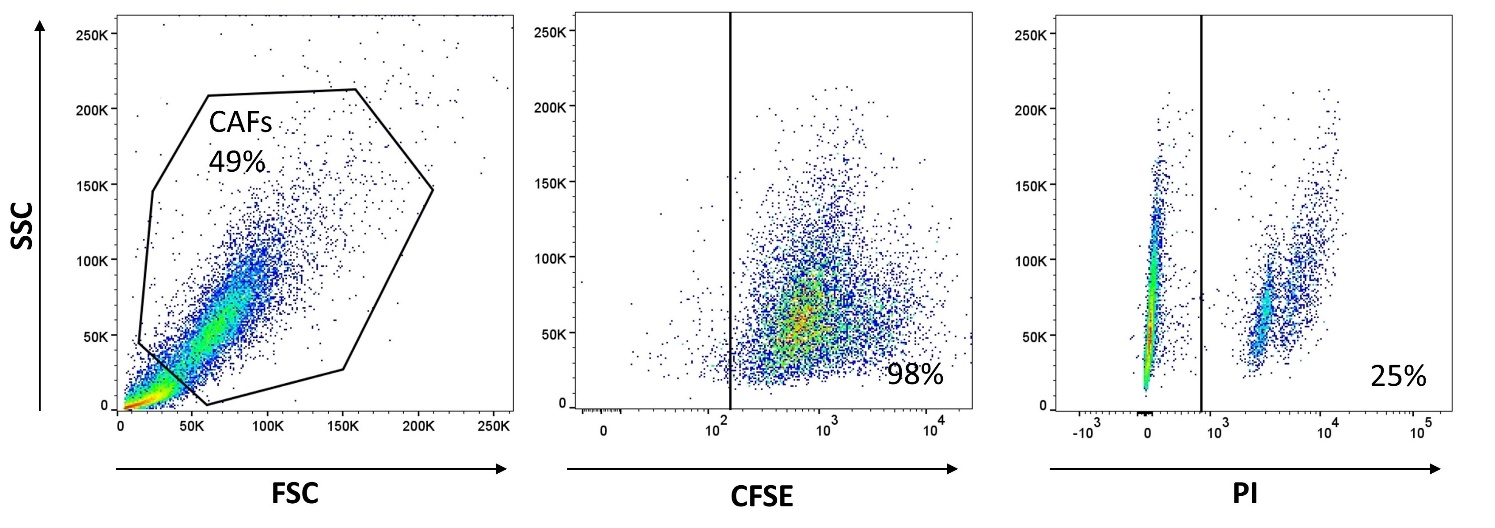


**Supplementary figure 1. Gating strategy for NK cell-mediated killing of CAFs.** CAFs were gated according to their scatter properties (FCS-A vs SCC-A). The PI positive cells from the CFSE+ population were considered dead target cells.


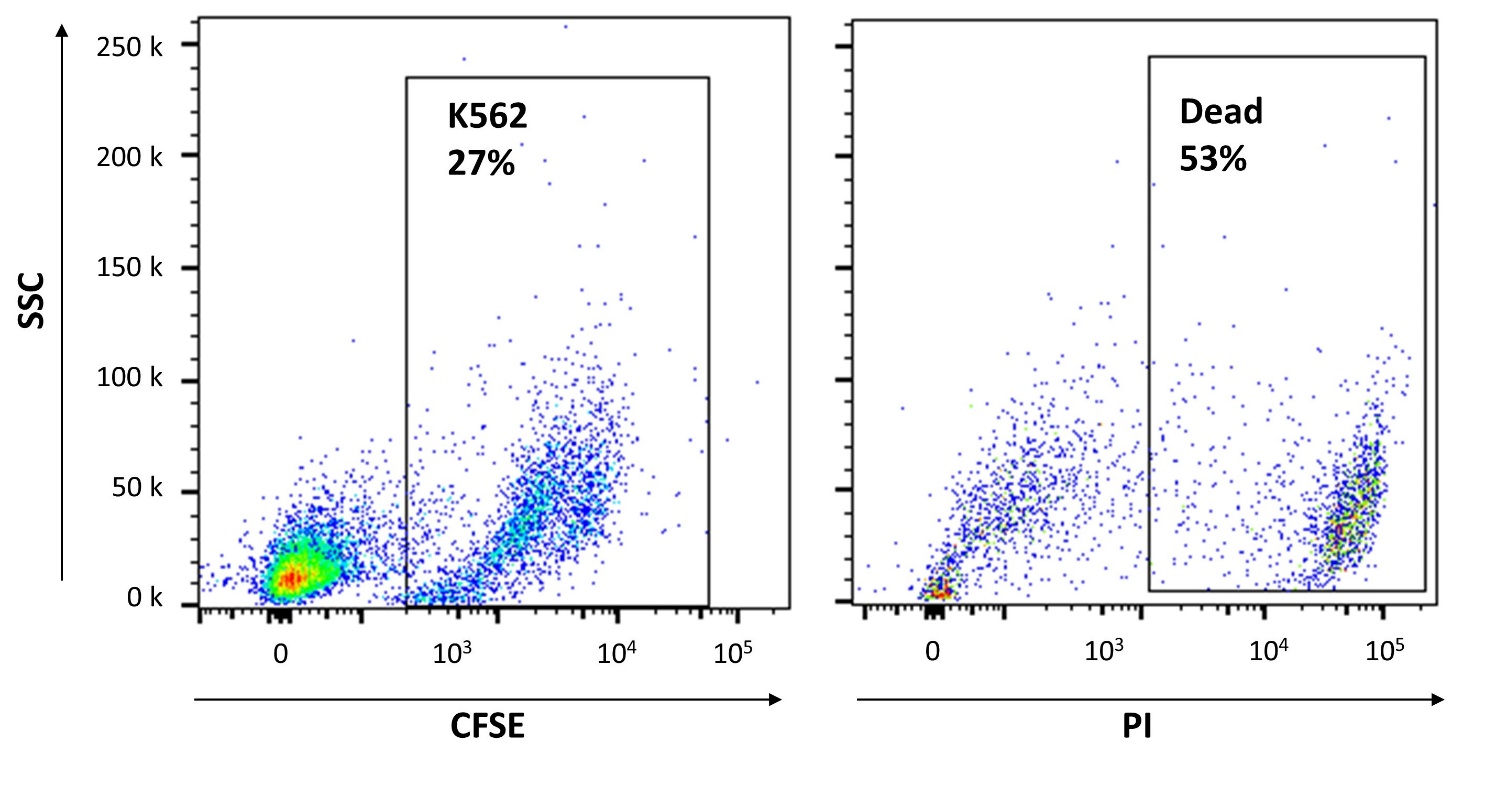


**Supplementary figure 2. Gating strategy for effects of CAFs on NK cell cytotoxic activity.** K562 cells were gated according to CFSE+ and SSC (CFSE vs SSC-A). PI positive cells from CFSE+ population were considered killed target cells.

**
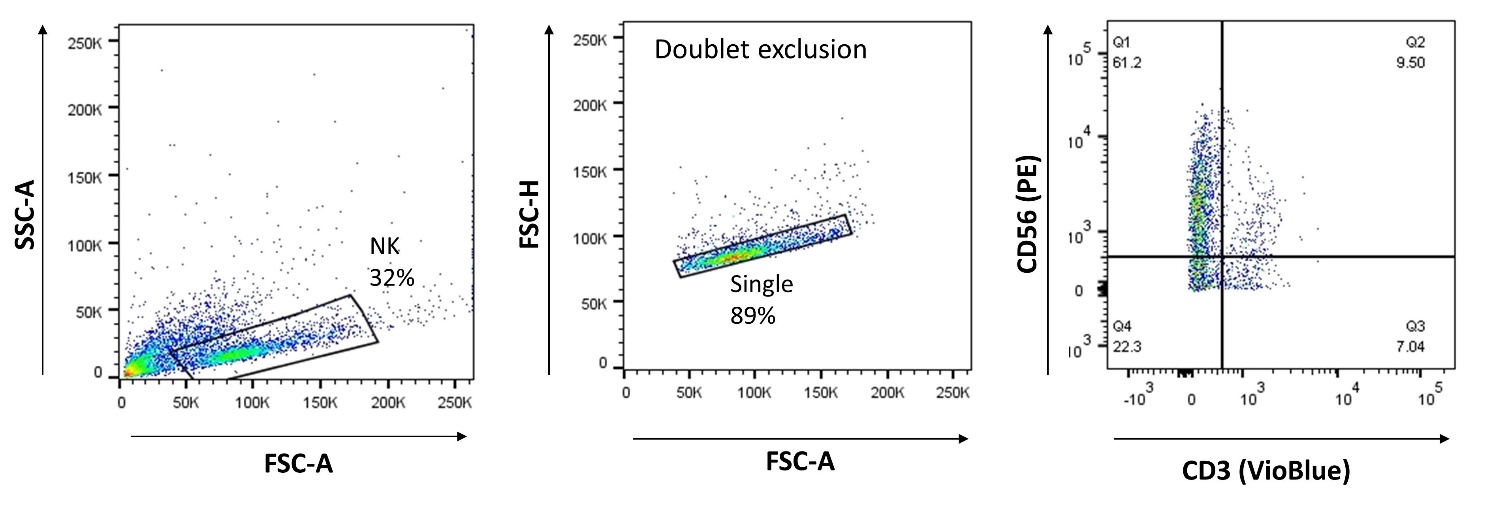
**

**Supplementary figure 3. Gating strategy for effects of CAFs on intracellular NK activation markers.** NK cells were gated according to their scatter properties (FSC-A vs SSC-A) to exclude platelets and cell debris. CD56+CD3- cells from the single cell population (FCS-A vs FSC-H) were analyzed for fluorescence of selected markers.

**
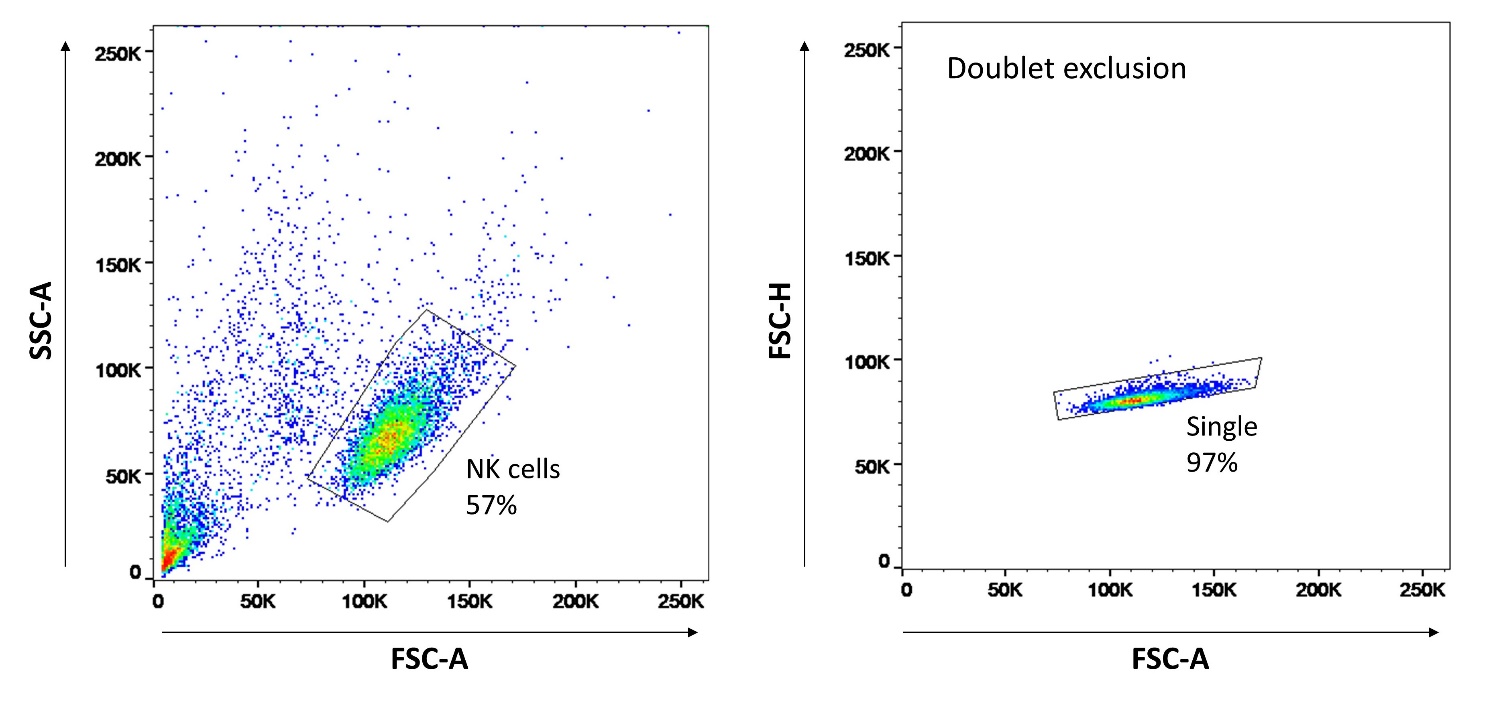
**

**Supplementary figure 4. Gating strategy for effects of CAFs on NK surface receptor expression.** NK cells were gated according to their scatter properties (FSC-A vs SSC-A) and doublet exclusion (FCS-A vs FSC-H).

**
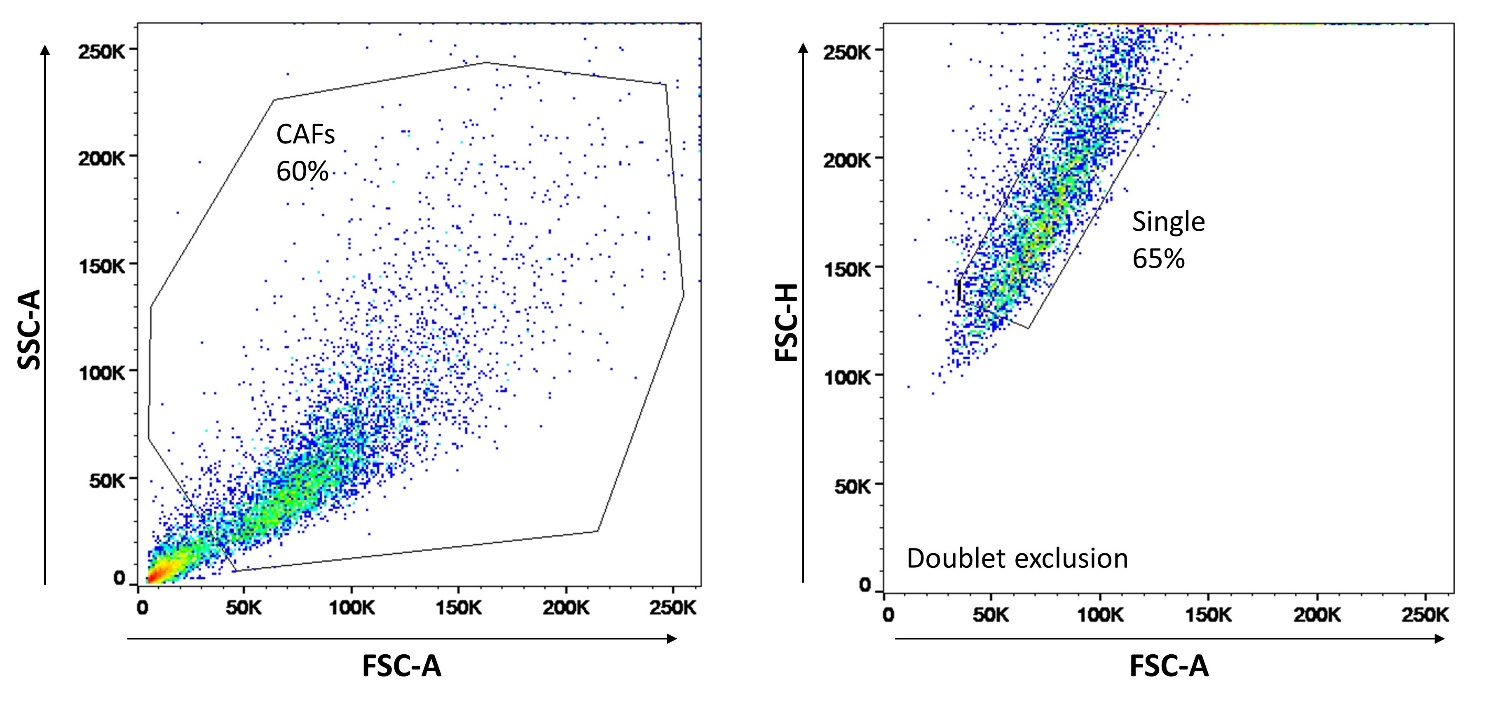
**

**Supplementary figure 5. Gating strategy for effects of radiation on CAF surface expression of checkpoint molecules**. CAFs were gated according to their scatter properties (FSC-A vs SSC-A) and doublet exclusion (FSC-A vs FSC-H).
